# 纺锤体组装检查点在肺癌中的研究进展

**DOI:** 10.3779/j.issn.1009-3419.2023.101.13

**Published:** 2023-04-20

**Authors:** QIN Xinchen, ZHANG Yao, YU Haijie, MA Lijuan

**Affiliations:** 999078 澳门，澳门科技大学中药质量研究国家重点实验室，埃尔文内尔博士生物物理与创新药物实验室; Dr. Neher's Biophysics Laboratory for Innovative Drug Discovery, State Key Laboratory of Quality Research in Chinese Medicine, Macau University of Science and Technology, Macau 999078, China

**Keywords:** 纺锤体组装检查点, 非整倍体, 有丝分裂, 有丝分裂检查点复合体, Spindle assembly checkpoint, Aneuploidy, Mitosis, Mitotic checkpoint complex

## Abstract

纺锤体组装检查点（spindle assembly checkpoint, SAC）是细胞正确进行有丝分裂（mitosis）的一种保护机制，以防止有丝分裂中后期染色体动粒（kinetochore）存在未附着或不正确附着微管的情况下继续有丝分裂，从而避免整条染色体的增加或丢失而产生非整倍体（aneuploidy）细胞。非整倍体以及SAC组成蛋白的表达改变是不同癌症种类，包括肺癌的共同特征之一。因此，SAC是一种潜在的肺癌治疗新靶点。目前，SAC上游组分蛋白单极纺锤体蛋白激酶1（monopolar spindle 1, MPS1）小分子抑制剂已有5种进入临床试验。本文介绍了SAC的生物学功能，总结了SAC组分蛋白在多种癌症中的表达异常和MPS1抑制剂的研究进展，期望对未来开发靶向SAC组分的肺癌治疗策略提供参考。

## Abstract (en)

Spindle assembly checkpoint (SAC) is a protective mechanism for cells to undergo accurate mitosis. SAC prevented chromosome segregation when kinetochores were not, or incorrectly attached to microtubules in the anaphase of mitosis, thus avoiding aneuploid chromosomes in daughter cells. Aneuploidy and altered expression of SAC component proteins are common in different cancers, including lung cancer. Therefore, SAC is a potential new target for lung cancer therapy. Five small molecule inhibitors of monopolar spindle 1 (MPS1), an upstream component protein of SAC, have entered clinical trials. This article introduces the biological functions of SAC, summarizes the abnormal expression of SAC component proteins in various cancers and the research progress of MPS1 inhibitors, and expects to provide a reference for the future development of lung cancer therapeutic strategies targeting SAC components.

真核生物，包括人的体细胞通过有丝分裂（mitosis）的方式增殖，有丝分裂包括前期、中期、后期和末期4个阶段。在有丝分裂前中期，染色单体整齐地排列在赤道板上，姐妹染色单体的动粒（kinetochore）各自被细胞两极的纺锤体发出的微管（microtubule）附着后，细胞开始进入分裂后期，姐妹染色单体被微管拉向两极，分裂成两个完全一样的细胞。染色体在中后期的这种精确分离由多种机制调控，其中主要包括纺锤体组装检查点（spindle assembly checkpoint, SAC）。SAC会通过调节动粒-微管连接来防止染色体的错误分离^[1]^。

SAC功能缺陷会导致染色体不稳定性（chromosomal instability, CIN）和染色体不正确分离造成非整倍体（aneuploidy），即异常的染色体数目。大多数肿瘤都具有非整倍体性的特点 ^[2]^。非小细胞肺癌（non-small cell lung cancer, NSCLC）是一种容易复发的恶性肿瘤，5年生存率约为15%，约有60%的NSCLC患者的肿瘤呈非整倍体性，小细胞肺癌（small cell lung cancer, SCLC）的非整倍体发生率为77.8%，高于其他组织学类型的癌症^[3]^。因此，专门针对非整倍体细胞的治疗干预是肺癌治疗的一种很有潜力的策略，以SAC组分蛋白作为靶点设计和开发治疗癌症的新药物是抗癌药物研发的新机遇。本文整理了目前对SAC作用机制的研究及其相关蛋白的协调作用和它们与肺癌之间的关系，这为全面了解肿瘤发生机制和以SAC组分蛋白为抗肿瘤靶点的药物研发提供了理论基础。

## 1 SAC概述

SAC经典成分包括有丝分裂停滞缺陷蛋白家族（mitotic arrest deficient, MAD）的MAD1、MAD2和BUBR1（酵母、线虫和植物中称为MAD3）、苯并咪唑出芽抑制解除同源物蛋白家族（budding uninhibited by benzimidazoles, BUB）的BUB1和BUB3以及其他组分，比如单极纺锤体蛋白激酶1（monopolar spindle 1, MPS1）、细胞分裂周期蛋白20（cell-division cycle protein 20, CDC20）和动粒受体，例如Rod-Zwilch-ZW10（RZZ）复合物^[4]^。动粒未附着微管时，MPS1会招募其他组分到未附着的动粒上，MAD2、BUB1、BUBR1和CDC20会组合成有丝分裂检查点复合体（mitotic checkpoint complex, MCC），延迟后期的开始。当动粒全部正确附着后，SAC沉默，SAC依赖RZZ复合物从动粒处解离。

### 1.1 SAC的功能

SAC是一种监测机制，可确保有丝分裂中染色体的正确分离。SAC会检查到未附着微管的动粒发出“等待后期”的信号，单个未连接微管的动粒足以引起SAC的激活以延迟后期^[5]^，未连接微管的动粒数量与SAC信号的强度成正比^[6]^。如图1所示，当动粒未附着微管时，SAC会被激活催化MCC（BUBR1-BUB3-MAD2-CDC20）复合物的组装^[7,8]^。MCC的组装抑制APC/C（anaphase promoting complex/cyclosome）后期促进复合物/细胞周期体的激活以及其底物蛋白分离酶抑制蛋白Securin和Cyclin B1的泛素化^[9]^。姐妹染色单体仍然由着丝粒（centromere）处的黏连蛋白（cohesin）捆绑在一起，不会被分离酶Separase水解导致过早分离。CDC20是负责底物识别并相互作用的APC/C共激活子亚基，通过识别APC/C底物的D-box（destruction box）、KEN-box（Lys-Glu-Asn-box）和ABBA基序（KxxFxxYxDxxE基序）促进APC/C的激活，而MCC竞争结合这些基序，阻断底物与APC/C结合以此延迟后期^[10,11]^。当所有动粒都正确附着微管后，SAC沉默，MCC解聚，APC/C^CDC20^泛素化Securin，使分离酶活化破坏着丝粒黏连蛋白的凝聚力，促进姐妹染色体分离和有丝分裂的退出。

**图 1:**
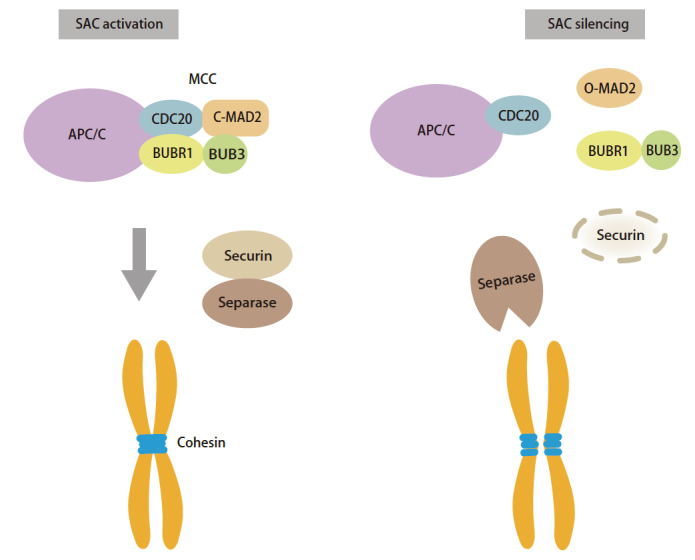
SAC的激活与沉默。 SAC激活时组装MCC拮抗APC/C活性，Securin抑制分离酶Separase活性，保护黏连蛋白（cohesin）凝聚力，姐妹染色单体仍结合在一起。SAC沉默后，MCC解离，APC/C泛素化Securin，分离酶破坏黏连蛋白，染色单体开始分离。

MCC通过两种途径抑制APC/C的活性——MAD2途径和BUBR1途径。MAD2能通过CDC20的MIM基序（MAD2-interacting motif）与之结合，催化MAD2-CDC20形成的关键是MAD2的两种构象异构体——O-MAD2（开放活性）和C-MAD2（封闭活性）^[12]^。这两种构象在MAD2的C末端区域结构上有所不同，称为“safety belt”^[13]^。MAD2这两种构象可以互相转换，主要通过O-MAD2与MAD1的结合转变为C-MAD2，再与CDC20结合^[14]^。激酶MPS1也会通过磷酸化MAD1和BUB1促进MAD2-CDC20的结合^[8,15,16]^（图2）。而BUBR1以独立于MAD2的方式直接抑制APC/C^CDC20^^[17]^。BUBR1通过ABBA基序和D-box与CDC20结合^[18]^，阻断CDC20上APC/C底物识别位点。BUBR1还阻碍UbcH10（APC/C特异性泛素结合酶E2）结合底物以抑制APC/C 泛素化活性^[18]^。

**图 2:**
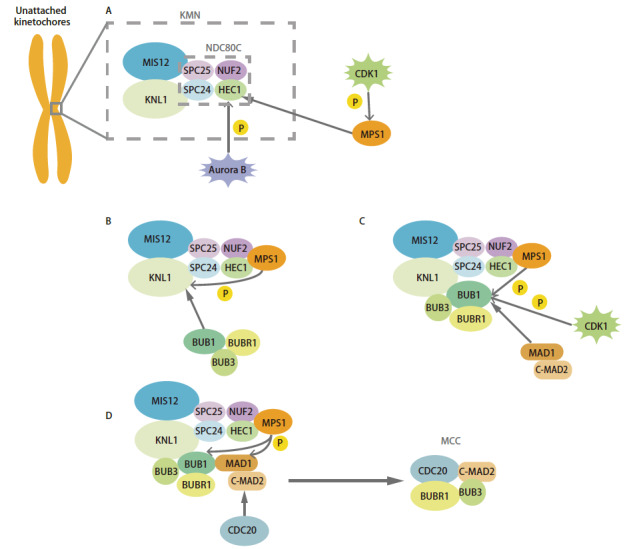
SAC的组装。动粒未附着微管时SAC被激活，MCC完成组装的过程。 A：激酶Aurora B磷酸化HEC1，CDK1磷酸化MPS1，促进MPS1向HEC1和NUF2的募集；B：MPS1磷酸化KNL1的MELT基序，促进BUB1、BUBR1和BUB3的募集；C：CDK1和MPS1磷酸化BUB1，刺激MAD1与BUB1的结合；D：MPS1磷酸化BUB1和MAD1，刺激C-MAD2与CDC20的结合，BUBR1-BUB3、MAD2、CDC20结合形成后期抑制复合物MCC。

### 1.2 SAC的组装

外动粒复合物KMN网络（Knl1-Mis12-Ndc80 network）是主要的动粒-微管结合部位，又是微管未结合时MCC组装的平台^[19]^，包含3个子复合体——MIS12复合体、NDC80复合体和KNL1复合体。NDC80复合物由SPC24、SPC25、NUF2和HEC1（酵母中称NDC80）蛋白组成。MPS1是一种双特异性激酶，又被称为酪氨酸/苏氨酸激酶（threonine and tyrosine kinase, TTK）或磷酸酪氨酸挑选的苏氨酸激酶（phosphotyrosine-picked threonine kinase, PYT）^[20]^。MPS1是SAC的上游组件也是核心成分，在确保染色体正确分离的过程中发挥着核心作用。MPS1通过自身N端片段与NDC80复合体的HEC1和NUF2成分结合从而被招募到外动粒上（图2A）^[21,22]^。虽然MPS1可以独立定位于动粒上，但Aurora B和CDK1可以促进MPS1的动粒定位。Aurora B磷酸化HEC1尾部增强MPS1与之结合，而CDK1通过磷酸化MPS1-S281促进MPS1与未附着微管的动粒结合^[21,23]^。MPS1的激酶活性有助于招募SAC其他组分蛋白^[24]^。KNL1（也被称为Blinkin，酵母Spc7同源物）在N端具有重复序列，称为MELT。有丝分裂期间MPS1会磷酸化KNL1的MELT片段促进BUB1-BUB3和BUBR1-BUB3的募集^[15]^（图2B）。BUB1的动粒募集独立于BUBR1，而BUBR1的募集严格从属于BUB1，BUB1会促进BUB3与磷酸化的KNL1结合^[25,26]^。除了在酿酒酵母中MAD1和MAD2的募集是相互依赖的，大多数生物中MAD1-MAD2复合物的动粒募集仅依赖于MAD1^[27,28]^。人类BUB1中的保守结构域1（conserved domain 1, CD1）直接与MAD1绑定，CD1中存在两个磷酸化位点（Ser459和Thr461），CDK1先磷酸化Ser459以允许MPS1随后磷酸化Thr461，促进MAD1与BUB1结合和SAC信号传导（图2C）。在酵母和人之间负责与磷酸化BUB1结合的MAD1的RLK基序是保守的^[29]^。最终，BUBR1-BUB3、MAD2与CDC20结合形成后期抑制复合体MCC拮抗APC/C的作用（图2D）。

## 2 SAC与肺癌的关系

导致染色体数目异常（非整倍性）的CIN是癌症的标志之一。有丝分裂细胞中的非整倍性可能由许多缺陷引起，包括纺锤体多极性、动粒-微管附着缺陷、微管动力学紊乱、染色单体凝聚力缺陷和SAC功能受损。弱化的SAC可能允许细胞在存在未连接或未对齐的染色体的情况下进入后期^[30]^。因此，SAC的功能缺陷是有丝分裂过程中非整倍体产生的一个主要因素^[31]^。

NSCLC是目前最常见的恶性肺肿瘤之一，患者生存率较低^[32]^，在64%的NSCLC细胞中发现了染色体非整倍性，非整倍体肿瘤患者的生存期短于近二倍体肿瘤患者^[33]^。既往研究^[34]^表明，SAC组分蛋白的异常会提高患癌风险，比如Bub3^+/-^小鼠表现出对化学诱导的肺部肿瘤敏感性增加的趋势。BubR1^+/-^小鼠胚胎成纤维细胞在SAC激活中有缺陷，Securin和CDC20显著减少，接触致癌物后迅速发展为肺癌或肠腺癌^[35]^。

我们整理了SAC在肺癌中的研究结果。致癌基因KIAA0101与UbcH10协同导致SAC组分蛋白BUBR1、MAD2和Cyclin B在NSCLC肿瘤组织中的表达显著低于癌旁组织，诱导肿瘤CIN和恶性增殖^[36]^。在体内，无论是沉默UbcH10基因还是同时沉默UbcH10和KIAA0101，都能够抑制肿瘤生长。共同沉默这两个基因可以产生更好的抑癌效果，表明KIAA0101与UbcH10可以共同靶向治疗NSCLC。在Eml4-Alk（致癌基因）小鼠中的研究发现，在肺上皮细胞过表达MAD2，小鼠肿瘤发展更快。此外，这些小鼠还表现出了高度CIN和T细胞衰竭的特征^[37]^。这可能是MAD2过表达引起的非整倍体性有助于制造一个免疫抑制微环境，促进肿瘤的发生和发展。模拟马赛克杂色非整倍体综合征（mosaic variegated aneuploidy, MVA）患者杂合BUBR1突变小鼠BubR1^+/L1002P^和BubR1^+/-^小鼠品系发生淋巴癌的概率增大，暴露于致癌物DMBA（7,12-dimethylbenz(a)anthracene）时也表现出肺部肿瘤增大的现象^[38]^。

肺癌化疗药物顺铂常在治疗中出现耐药性。经过分析A549细胞产生顺铂耐药前后的转录组数据，发现在顺铂耐药的A549细胞中BUB1、BUB1B（编码BUBR1）、CDK1、KIAA0101高表达^[39]^，表明这些基因可能会促进NSCLC的化学耐药性。这为后期联合用药降低肺癌治疗中的耐药性提供了参考思路。

## 3 SAC在癌细胞中的突变和异常表达

尽管编码SAC蛋白的基因在人类癌症中很少发生突变，但SAC蛋白在许多癌细胞中过度表达，这种异常表达可能是癌症的起因，也可能是癌症发展的结果（表1）。这些异常的表达可能导致癌细胞的增殖和生长，与患者的生存期和预后相关，因此，对于这些异常的SAC蛋白表达的研究也是癌症研究热点之一。

**表1:** SAC蛋白在肺癌中的表达

SAC protein	Expression level	Evidence	Reference
MPS1	Overexpression	Immunohistochemistry, chemical labeling, quantitative proteomic screening	[43,45]
BUB1	Overexpression	Bioinformatics analysis, Immunological analysis	[48,49]
BUBR1	Overexpression	Bioinformatics analysis, RT-PCR	[51,52]
BUB3	Overexpression	Immunological analysis	[49]
MAD1	Lower expression, missense mutation	Immunohistochemistry, RT-PCR-SSCP analysis	[54,56]
MAD2	Overexpression	Agarose electrophoresis and quantitative real-time-PCR	[58]
CDC20	Overexpression	Immunohistochemistry	[61]

RT-PCR: reverse transcription-polymerase chain reaction; SSCP: single-stranded conformation polymorphism.

### 3.1 MPS1

MPS1是SAC的核心成分，在SAC信号通路中位于上游，它的表达量的异常会影响SAC的功能。然而，在多种癌症中发现MPS1高表达，包括肺癌、胶质母细胞瘤、乳腺癌、结肠癌、肝细胞癌等^[40⇓-42]^。在肺腺癌患者的组织标本中，发现TTK（MPS1）和MAD2L1的表达水平相较于癌旁正常肺组织明显增加^[43]^。多项研究^[44,45]^表明MPS1的表达水平在多种肺癌中高表达，并且与肿瘤分级和不良预后呈正相关。这些研究表明，MPS1抑制剂可能成为治疗包括肺癌在内的多种癌症的潜在药物，为临床治疗提供更为广阔的应用前景。

### 3.2 BUBs

首次报道的SAC基因的种系突变疾病称为马赛克杂色非整倍体综合征。马赛克杂色非整倍体综合征是一种罕见的隐性疾病，其特征是生长迟缓、小头畸形、儿童癌症和同一个体染色体数目不同的镶嵌现象。在5个患有马赛克杂色非整倍体综合征的家庭中发现了BUB1B（编码BUBR1的基因）突变，共发现9种突变，其中6种突变位于激酶结构域^[46]^。这些数据首次将SAC基因的种系突变与人类疾病联系起来，支持了非整倍性与癌症发展之间的关系。

在肺癌细胞中，BUBs的突变罕见，通常发生过表达。目前仅在3种原发性肺癌细胞中发现BUB1外显子突变^[47]^。在肺腺癌中BUB1、BUB3和BUBR1的表达显著高于正常组织，患者BUB1、BUB3和BUBR1的转录水平显著高于健康人群。此外，BUB1、BUB3和BUBR1的表达与患者总生存期呈负相关，表明BUB1、BUB3和BUBR1可能是肺腺癌的生物标志物^[48⇓⇓⇓-52]^。BUBR1敲低的小鼠可以延缓肺部肿瘤的进展，延长总生存期^[51]^。只在少数癌症中BUBs的表达下调，比如BUBR1在绝大多数急性髓性白血病患者中下调^[53]^。

### 3.3 MADs

在Nomoto等^[54]^的报道中，约40%的人类肺癌细胞出现有丝分裂检查点损伤，通过检查49份肺癌样本首次发现MAD1在癌症中的突变。编码MAD1和MAD2基因的错义突变会增加患者肺癌易感性^[55]^，这可能是由于MAD1、MAD2功能减弱使SAC降低所致。在调查云南40名矿工和20名农民的肺癌样本中，研究^[56]^发现肺癌组织中MAD1的表达水平明显低于正常肺组织，表明MAD1的表达下调与肺癌的发生有关。MAD2过表达已在多种不同的癌症中被报道，包括肺癌、结肠癌、肝细胞癌、前列腺癌、乳腺癌等^[57]^，除卵巢癌外，MAD2表达增加与癌症全因死亡率和复发风险增加有关。MAD2的过表达在不同肺癌中程度也不同，SCLC患者MAD2表达明显高于NSCLC患者^[58]^，表明MAD2可作为诊断SCLC的特异性标志物。MAD2的过表达与肺腺癌的总生存期与无复发生存期呈负相关^[59]^。综上所述，MAD2可作为肺癌的潜在治疗靶点。

### 3.4 CDC20

CDC20通常是一种促癌基因，与DNA损伤修复过程受损和细胞凋亡有关。CDC20在乳腺癌、卵巢癌、骨肉瘤、肝细胞癌等多种癌症干细胞和恶性肿瘤中过表达。作为许多类型癌症的预后标志物，CDC20过表达可预测肿瘤高级别、晚期和预后不良^[60]^。有研究^[61]^表明，CDC20高表达可能是NSCLC的潜在预后标志物，它与MAD2表达之间存在显著相关性，且存在性别特异性——男性特异性，CDC20高表达使NSCLC患者总生存期显著缩短。因此，研发CDC20小分子抑制剂可能是治疗肿瘤的新途径，但目前仅报道过一种CDC20抑制剂——Apcin，但其抗细胞增殖的能力较弱，尚未进入临床试验阶段。

## 4 SAC MPS1小分子抑制剂

SAC组分蛋白在多种癌症中发现过表达，MPS1作为SAC的上游成分，研发靶向MPS1激酶的小分子抑制剂可作为治疗癌症的新药研发策略。Cincreasin是第一个报道的MPS1抑制剂，它是在出芽酵母中对140,000个小分子药物进行基于表型的遗传筛选中被发现的^[62]^。目前，多个MPS1小分子抑制剂被报道，其中5个抑制剂已进入临床试验阶段——BAY-1217389、BAY-1161909、CFI-402257、S81694和BOS172722（表2）。

**表2:** MPS1抑制剂的临床试验

Drug name	Clinical trials	Phase	Disease	Interventions
BAY-1217389	NCT02366949	I	Advanced solid malignancies	Combination with Paclitaxel
BAY-1161909	NCT02138812	I	Advanced solid malignancies	Combination with Paclitaxel
CFI-402257	NCT03568422	II	Advanced/metastatic HER2-negative breast cancer	Combination with Paclitaxel
	NCT02792465	I	Advanced solid cancers, breast cancer	Combination with Fulvestrant
S81694	NCT03411161	II	Metastatic breast cancer	Combination with Paclitaxel
	MPSA-153-001	II	Hepatocellular carcinoma	Monotherapy
BOS172722	NCT03328494	I	Advanced nonhematologic malignancies	Combination with Paclitaxel

HER2: human epidermal growth factor receptor 2.

MPS1小分子抑制剂BAY-1217389和BAY-1161909由德国拜耳制药公司（Bayer Pharma AG）研发，可以抑制MPS1激酶活性，半抑制浓度（half maximal inhibitory concentration, IC_50_）低于10 nmol/L^[63]^。这两种抑制剂可以拮抗Nocodazole诱导的SAC激活，导致肿瘤细胞死亡。在裸鼠中移植紫杉醇耐药性NCI-H1299细胞系中，BAY-1217389与紫杉醇联合给药36 d后，BAY-1217389明显增强了紫杉醇的疗效。而在裸鼠中移植来源紫杉醇耐药肺癌患者肿瘤模型LU387中，BAY-1161909与紫杉醇联合给药会明显抑制肿瘤的生长。BAY-1217389和BAY-1161909与紫杉醇联用不会增加毒性并且可能可以克服耐药性^[63]^。目前，这两种抑制剂与紫杉醇联合使用正在进行实体瘤I期临床试验（NCT02138812和NCT02366949），以改进剂量确定最大耐受剂量^[64]^。

CFI-402257是一种具有口服活性、高选择性的MPS1小分子抑制剂^[65]^。CFI-402257在多种人类肺癌细胞和小鼠肺癌细胞中均显示出显著的抗肿瘤活性，可能与丝裂原活化蛋白激酶（mitogen-activated protein kinase，MAPK）通路有关^[66]^。未来可以将CFI-402257和MAPK抑制剂联用观察是否可以增强抗癌活性。除肺癌外，CFI-402257对CDK4/6抗性乳腺癌有治疗作用^[67]^，BAY-1217389和CFI-402257与替莫唑胺（Temozolomide, TBM）结合使用可能有助于多形性胶质母细胞瘤患者克服替莫唑胺耐药性^[68]^。目前CFI-402257正在进行晚期实体瘤的I期临床试验（NCT02792465），与紫杉醇联用已进入治疗晚期/转移性HER2阴性乳腺癌的I期/II期临床试验（NCT03568422）。

S81694目前结构未公开，但安全且耐受性好。在对晚期转移性实体瘤患者的I期试验中，共有39例患者，2例为肺癌患者，18例患者为发生肿瘤肺部转移，每周静脉注射耐受且剂量递增至每周135 mg/m^2^未达到最大耐受剂量^[69]^（NCT03411161）。根据I期确定的剂量，S81694与紫杉醇联用治疗转移性乳腺癌II期试验已经完成但尚未公布。在另一I期/II期试验（MPSA-153-001）的I期试验结果中，S81694在复发或难治性不可切除肝细胞癌患者中显示出临床疗效，其中1例患者在接受3次既往全身治疗后，肝脏靶病灶大小缩小了27%。

BOS172722单独治疗会诱导癌细胞凋亡，特别是在SAC活性受损的高度增殖性三阴性乳腺癌细胞系中。BOS172722和紫杉醇联用会增强抗肿瘤效果^[70]^。该药物和紫杉醇联用已进入治疗晚期非血液系统恶性肿瘤的临床I期试验（NCT03328494）。

目前的临床试验中，MPS1抑制剂大多与紫杉醇联用，进行MPS1抑制剂单药治疗的试验很少，也许与癌细胞对MPS1抑制剂敏感度不够有关。在Yael Cohen-Sharir和他同事的研究^[71]^中发现非整倍体癌细胞对MPS1抑制剂或其他SAC组分的敲低的敏感程度低于整倍体癌细胞对MPS1抑制剂的敏感性，具有更高的抵抗力，但后期会累积严重的细胞畸变导致细胞死亡。KIF18A是一种有助于CIN肿瘤细胞增殖的蛋白，但对于近二倍体细胞不是必需蛋白^[72]^，它的过表达与癌症的不良预后有关^[73]^。KIF18A单独过表达对细胞活力和增殖的影响很小，但它的敲低使非整倍体细胞对MPS1抑制剂MPI-0479605和Reversine敏感^[71]^。MPI-0479605和Reversine目前未进入临床试验阶段，这个发现对将来开发更多的MPS1抑制剂的临床试验具有指导意义，也为MPS1抑制剂与KIF18A抑制剂的联合用药以减少耐药性的研究提供了基础。后期可以开展SAC其他组分的抑制剂与KIF18A抑制剂的联合用药的研究，但这种联合用药是否会增强细胞毒性还是未知的。

## 5 总结

非整倍体是癌细胞的特征之一，有丝分裂期间的染色体错误分离可能导致染色体非整倍性——其中一个或多个染色体没有正确分离到子代细胞中。这种错误在正常细胞中很少见，但在肿瘤发展过程中频繁地发生。高水平的非整倍性与患者的不良预后有关。高水平的内源性CIN与多种癌症并发症和治疗耐药性的增加相关。在非整倍体癌细胞中，有些蛋白的表达与正常细胞不同，未来的工作可以更针对这些蛋白进行深入研究。

SAC组分的表达在多种癌症类型中发生改变，少数病例携带突变的SAC基因，而不同癌症的SAC组分表达的变化是不同的。MPS1抑制剂的开发可以根据作用机制研究，设计新策略以提高其抗肿瘤药效。MPS1抑制剂对实体瘤有疗效。该篇综述总结了SAC的组分及其组装的机制，各组分蛋白在不同癌细胞包括NSCLC的表达异常情况，并进一步总结了以SAC组分特别是MPS1为靶标的癌症治疗的小分子抑制剂的研究进展，这为以SAC作为靶标治疗肺癌的研究提供有价值的参考。此外，SAC靶向药物与其他药物协同作用值得进一步探索，这将对未来的药物开发提供新思路。
